# 

**Published:** 1885-11

**Authors:** 


### CONTENTS.

PROCEEDINGS.

American Dental Association........................................................ 533
British Dental Association........................................................ 568

CORRESPONDENCE.

On Local Anæsthetic—Cocaine........................................................ 577

SALMAGUNDI........................................................ 578

EDITORIAL.

Ohio State Dental Society........................................................ 582

